# Perspectives of People Living with HIV on Access to Health Care: Protocol for a Scoping Review

**DOI:** 10.2196/resprot.5263

**Published:** 2016-05-18

**Authors:** Shabnam Asghari, Allison Maybank, Oliver Hurley, Hilary Modir, Alison Farrell, Zack Marshall, Claire Kendall, Sharon Johnston, Matthew Hogel, Sean B Rourke, Clare Liddy

**Affiliations:** ^1^ Primary Healthcare Research Unit Department of Family Medicine, Faculty of Medicine Memorial University of Newfoundland St. John's, NL Canada; ^2^ School of Pharmacy Memorial University of Newfoundland St. John's, NL Canada; ^3^ Health Sciences Library Faculty of Medicine Memorial University of Newfoundland St. John's, NL Canada; ^4^ Division of Community Health and Humanities Faculty of Medicine Memorial University of Newfoundland St. John's, NL Canada; ^5^ C.T. Lamont Primary Health Care Research Centre Bruyere Research Institute Ottawa, ON Canada; ^6^ Department of Family Medicine University of Ottawa Ottawa, ON Canada; ^7^ Ottawa Hospital Research Institute Ottawa, ON Canada; ^8^ Institute of Clinical Evaluative Sciences Toronto, ON Canada; ^9^ Bruyere Research Institute Department of Family Medicine, Faculty of Medicine University of Ottawa Ottawa, ON Canada; ^10^ Ontario HIV Treatment Network Toronto, ON Canada; ^11^ Li Ka Shing Knowledge Institute St. Michael's Hospital Toronto, ON Canada; ^12^ Department of Psychiatry University of Toronto Toronto, ON Canada

**Keywords:** HIV, HIV Infections, Attitude to Health, Patient Satisfaction, Perspective, Health Services Accessibility, Health Services/Utilization, Access to Health Care

## Abstract

**Background:**

Strategies to improve access to health care for people living with human immunodeficiency virus (PLHIV) have demonstrated limited success. Whereas previous approaches have been informed by the views of health providers and decision-makers, it is believed that incorporating patient perspectives into the design and evaluations of health care programs will lead to improved access to health care services.

**Objective:**

We aim to map the literature on the perspectives of PLHIV concerning access to health care services, to identify gaps in evidence, and to produce an evidence-informed research action plan to guide the Living with HIV program of research.

**Methods:**

This scoping review includes peer-reviewed and grey literature from 1946 to May 2014 using double data extraction. Variations of the search terms “HIV”, “patient satisfaction”, and “health services accessibility” are used to identify relevant literature. The search strategy is being developed in consultation with content experts, review methodologists, and a librarian, and validated using gold standard studies identified by those stakeholders. The inclusion criteria are (1) the study includes the perspectives of PLHIV, (2) study design includes qualitative, quantitative, or mixed methods, and (3) outcome measures are limited to patient satisfaction, their implied needs, beliefs, and desires in relation to access to health care. The papers are extracted by two independent reviewers, including quality assessment. Data is then collated, summarized, and thematically analyzed.

**Results:**

A total of 12,857 references were retrieved, of which 326 documents were identified as eligible in pre-screening, and 64 articles met the inclusion criteria (56% qualitative studies, 38% quantitative studies and 6% mixed-method studies). Only four studies were conducted in Canada. Data synthesis is in progress and full results are expected in June, 2016.

**Conclusions:**

This scoping review will record and characterize the extensive body of literature on perspectives of PLHIV regarding access to health care. A literature repository will be developed to assist stakeholders, decision-makers, and PLHIV in developing and implementing patient-oriented health care programs.

## Introduction

Within the topic of access to health care for people living with human immunodeficiency virus (PLHIV) there are four key ideas which are very important to the conception and planning of research in this area. These ideas are used to guide research in an effective manner to be able to produce the best results and initiate change. These ideas are (1) efforts to improve health care will be wasted unless they reflect what patients want from the service [1], (2) access is a major concern in health care policy and is one of the most frequently used words in discussions of the health care system [2], (3) engagement in care is vital for the prognosis of PLHIV or acquired immune deficiency syndrome (AIDS), and to reduce the transmission of HIV [3], and (4) access and delivery of health care services to PLHIV is challenging and requires a characterization of these people and their perspective of access to health care [4-6].

### Access to Health Care for People Living with HIV

In developed countries, the number of PLHIV is increasing. With advances in HIV treatment and health care services, PLHIV are living longer, necessitating a wider range of preventive, acute, and long-term health care services to meet the needs of PLHIV across their lifetimes [4,5]. The complexity of responses to meet the needs of PLHIV is heightened as HIV continues to disproportionally affect vulnerable populations.

Providing health care for a patient population that experiences complex needs requires societies to develop and sustain appropriate health care, as well as improve access to health care services that ensure engagement and ongoing retention in care [4,7-9]. The positive impact of accessible health care on the health and quality of life of PLHIV has been previously identified [4,7-9]. In a system such as Canada's (that provides universal health care), it is often assumed that there are few barriers to accessing care and treatment, such as antiretroviral therapies. Indeed, Canadian studies show that most people diagnosed with HIV have had encounters with health care providers within one month of their diagnosis [5]. However, there are many people with HIV who have not engaged with care to adequately monitor and treat infection [5]. Access to required services for HIV care also varies across jurisdictions, and many PLHIV report continued difficulty accessing health care services [5]. Previous studies have revealed barriers to access, disparities in health care delivery, and factors contributing to underutilization of services among this population [4]. In addition, there have been attempts to mitigate these barriers, including strategies such as eHealth or outreach programs to improve access and engagement in health care [7]. However, most of these studies were developed and conducted based on the perspective of health care decision-makers, while patients’ views of access to health care may differ from health professionals, managers, and policymakers [10]. Incorporating patients’ perspectives into the implementation and evaluation of health care programs is essential [11]. This study represents the first attempt to broadly and systematically identify, classify, and synthesize literature on PLHIV’s perspectives on access to health care.

### Concept of End-Users and Patients’ Perspectives

It is increasingly recognized that patients’ beliefs and desires influence their involvement in their own health, access to health care, and communication with their health care providers. Recent studies also emphasize that patients’ perceptions of their access to health care services should be taken into account when implementing health programs [12-14]. However, there is no unique consensus for patients’ perspectives that can span all conditions and all populations [10,11].

Most existing literature has documented patients’ perspectives by assessing patient satisfaction using questionnaires and surveys; others suggest first identifying a common definition for the end-users of the health care system, including patients, consumers, citizens, and the general public. These studies classify the end-users into two categories. The first group includes individuals *whose role is to provide a societal or lay perspective about health services/technologies* [15]; this category includes groups representing citizens and elected officials. The second category includes *those individuals directly affected by a given health condition or health service/technology* [15]. One study by Cayton suggests that these two categories represent different roles that individuals take on while engaging with health care services, and identifies these categories as two sides of the same coin [16].

Finally, consumer-perspective studies suggest visualizing issues through the eyes of the service users. These investigations include consumers’ insights and *experiential* evidence of the totality of features of a product, and consumer-stated satisfaction (or their implied needs) with the products. The challenge in these studies is the implicit assumptions of people’s interpretation of quality and their individual values [1,11].

### Concept of Access to Health Care

Access to health care is a complex concept that is usually measured using multiple dimensions, including the characteristics and expectations of health care providers, customers, or patients. A growing body of research has grouped these characteristics into the five A’s of access to health care (affordability, availability, accessibility, accommodation, and acceptability) [2]. Many investigators suggest distinguishing between the *potential of having access* and *gaining access,* by identifying whether people who require health care get into the health care system or not.

Individuals’ perceptions of their needs for health care, and their decisions to seek health care services, are the first steps in the process of gaining access to health care. Access to health care services suggests that a person distinguishes and accepts his or her needs for health care, consents to be a service user, recognizes resources, and is willing to use the services available [17]. This process is further influenced by demographic, socioeconomic, cultural, and environmental factors. Individuals’ anticipation and recognition of a health care service may differ from those of health care professionals [17]. In recent years, efforts to change people’s attitudes and behaviors regarding health care services have increasingly shifted to acknowledging the importance of the views of users and their demands in developing services. Some of these advances extend the concept of access (beyond physical access) to the development of remote access via electronic devices and the Internet [18].

### Scope of the Review

We aimed to summarize the perspectives of PLHIV concerning access to health care, as identified in previous studies. In order to provide a more comprehensive overview of the existing knowledge, a scoping review will be conducted instead of a systematic review. Findings from the scoping review will enable us to examine the extent, range, and nature of research activity on this topic, and to evaluate the value and feasibility of performing a full systematic review [19].

### Protocol of the Review

The standard process for this scoping review will include the stages suggested by Arksey and O’Malley, among others [19-23]. The draft protocol is being circulated to knowledge translation experts, systematic review methodologists, a librarian, clinicians, and decision-makers for their review, and the protocol will be modified as required. The quality of individual studies will also be assessed, scored, and synthesized to gauge the overall quality of studies being done [19,21-23].

### Stage 1: Identifying the Research Question

The research question for this review was developed by members of Advancing Primary Healthcare for People Living with HIV (LHIV Innovation Team), a team of researchers, primary and specialist health care providers, policy-makers, and HIV-positive people with experience accessing health care, aiming to improve the community-based care of PLHIV in Canada. Our research question is summarized as: What is the extent of knowledge on the perspectives of PLHIV about access to health care services?

### Goals and Aims

In response to the research question, the main goal of this review is to explore the depth, breadth, and quality of evidence about the perspectives of PLHIV regarding access to health care services internationally, with a particular focus on Canadian studies. The aims of this scoping review are to orient our research team, to provide evidence-based information for the team to conduct the research, and to advance the primary health care for PLHIV in Canada.

### Objectives

We have five main objectives in this study:

1. Map the literature on the perspectives of PLHIV about access to health care services.
2. Describe what is known about the perspectives of PLHIV regarding access to health care services, to identify the gaps in evidence, and to highlight research priorities based on these results.
3. Establish the themes of the perspectives of PLHIV regarding access to health care.
4. Prepare an evidence-based summary of Canadian and international research literature on the perspectives of PLHIV related to health care access.
5. Produce an action plan and a research agenda for the LHIV Innovation Team.

## Methods

### Participants

The participants that will be targeted include anyone living with HIV/AIDS. There will be no exclusion criteria based on age, sex, ethnicity, or geographic location, although sub-populations may be discussed separately depending on our findings.

### Outcomes

Outcomes will be restricted to any measures of PLHIV-stated satisfaction, implied needs, beliefs, and desires concerning access to health care. No restrictions on the type of health care services are to be applied to our search, including traditional and non-traditional services for the diagnosis and treatment of disease or the maintenance of health, in order to include all outcomes in the literature. To distinguish access from other attributes of health care services, including continuity of care and retention, the definitions suggested by Haggerty et al will be used [24,25].

### Stage 2: Identifying Relevant Studies

#### Selection of Search Terms

Significant terms from the research question will be selected, and a list of possible synonyms or alternate terms will be compiled. To find the best search terms, Medical Subject Heading (MeSH) terms, MeSH tree, and related words found in key words and references will also be searched.

#### Building Search Terms Strategy

To determine the best search strategy, different combinations of words will be tested across databases. The search will include an iterative process to refine the search terms through testing of different terms, and combining new terms, as new related citations are identified. The search will include a combination of MeSH and keywords searched in the title and abstract (tiab) fields. Search strategies will be modified for other databases as required.

#### Sources of Relevant Studies

To identify all sources of information, this review will begin with a comprehensive mapping of peer-reviewed publications referenced in electronic databases. The search for studies will be in the following databases, and will be guided by a librarian: EMBASE (1947 to May 5, 2014); MEDLINE vis PubMed (1946 to May 5, 2014); CINAHL (1937 to May 5, 2014); Cochrane (1993 to May 5, 2014); and PsycINFO (1880s to May 5, 2014).

Cited references of articles chosen for inclusion in the scoping review will also be searched, as well as additional sources, including the reference lists of included studies, searching ProQuest for PhD theses, contacting experts to request details of any known studies (eg, known Canadian researchers in this subject area), HIV Conferences and Symposia, and all sources identified in Multimedia Appendix 1.

#### Validation of Search Protocol

To validate the search protocol and calibrate our search strategy, the protocol will be tested on the gold standard studies and journals suggested by content experts. The eligibility criteria will be modified as required.

#### Directory of the Identified Studies

A directory of publications and grey literature will be created in Refworks [26]. To capture and tag the web-pages’ information, other reference manager software will also be used, including Zotero [27] and Mendeley [28]. The tagged webpages will then be imported into Refworks.

### Stage 3: Study Selection

Study selection will be an iterative process consisting of searching the literature, refining the search strategy, assessing the eligibility criteria, pre-screening and reviewing the full text of the literature for inclusion, and retaining only articles concerning PLHIV’s perspectives on access to health care.

#### Eligibility Criteria

Decisions about review process methodology will be undertaken by members of our team who will be blinded to the results of the studies in question, and who have expertise in health care services for PLHIV. Inclusion criteria will ensure a wide range of literature from varying resources, but only French and English articles will be used, as reviewers are fluent in these languages. To ensure a good standard of evidence and clinical relevance to the review, the types of articles found under exclusion criteria will not be included.

Inclusion criteria will include (1) literature from peer-reviewed journals, (2) grey literature, such as unpublished theses and reports from relevant websites, and (3) the use of only French and English articles for full-text review. Exclusion criteria will include (1) audits or anecdotal information, (2) research at the planning stage (although this will be included in the research directory), (3) pilot studies, (4) undergraduate and MSc dissertations, (5) book reviews, and (6) policy analyses.

Qualitative and quantitative studies will be examined. Qualitative studies will include any kind of qualitative study (eg, phenomenology, ethnography, grounded theory, historical and case studies) as identified by the authors. Quantitative studies encompass reviews (systematic reviews, meta-analyses, narrative reviews, scoping reviews), observational studies (cohort, case control, cross-sectional studies, case series, case reports), and interventional studies (field trials, randomized controlled trials, community trials, quasi-experimental).

#### Study Selection Process

##### Step 1: Pre-screening

The titles and abstracts of all articles identified during database searches will be examined by a trained undergraduate student to evaluate eligibility, after duplicates are removed. Studies will be considered unrelated if the articles and abstracts are not related to the search subject, or if the articles are commentaries or editorials. The number of all articles deemed eligible by title and abstract will be recorded for further reference, but only French and English articles are subject to full-text reviews.

##### Step 2: Verification of Results

A random sample of 5% of the articles that are excluded by title and abstract will then be re-examined by one of the two reviewers to ensure that all relevant articles are considered. If more than 5% of the sample is found to be relevant, all excluded articles will be re-examined.

##### Step 3: Full Text Review

The full texts of retained references will be linked to the PLHIV Perspective Directory in Refworks using the Memorial University Library Services. The number of articles without full texts will also be recorded for further reference. Before commencing the full text review and data extraction, a calibration exercise will be undertaken. Two independent reviewers will be assigned a random selection of 5% of included citations. The eligibility criteria will then be modified if the agreement between the two reviewers is low (κ<0.5). The reviewers will also screen the remainder of the citations and discrepancies will be resolved by a third reviewer.

### Stage 4: Data Extraction

A data extraction tool in Excel will be prepared for data abstraction to systematically collect data from identified articles. The tool will be designed to extract information on the citation type (eg, original research), country, date of study, methodological aspects of the study, design, characteristics of the participants, participants’ perspectives on access to health care, and the quality of the study. The data extraction tool will be assessed on a random sample of 10 articles. The data extraction tool will also be revised iteratively, as required. Two trained graduate students will independently review and extract the information.

### Stage 5: Collating, Summarizing and Reporting the Results

Collating and summarizing results will include a descriptive summary of the number of identified articles, and an interpretive synthesis using the Arksey and O’Malley framework [19]. It is anticipated that the identified studies will incorporate different methodologies; qualitative and quantitative analyses will then be undertaken. The quality of original studies (both quantitative and qualitative) will be evaluated using a scoring system for Systematic Evaluation of Mixed Studies Review [23]. The quality of the screened articles will be graded, not with the intention of excluding the poorer studies or weighting the studies, but rather to identify the overall quality of studies in the sample. The studies and their characteristics will also be summarized. The frequency of studies will be reported by the study design, including place and date of publication, quality score, identified outcomes (PLHIV’s perspectives on access to health care), the positive and negative perspectives, and the PLHIV’s reported barriers and facilitators to accessing health care.

Quantitative studies will be reviewed and evaluated using a descriptive summary of key findings (eg, PLHIV’s reported facilitators and barriers, PLHIV’s views, satisfaction, attitudes, and opinions on access to health care) as well as the measurement tools (see Multimedia Appendix 2). Qualitative studies will be reviewed and evaluated by identifying the key findings and themes (eg, PLHIV’s reported barriers and facilitators, PLHIV’s views on access to health care). The findings will then be presented as an initial concept and further broken down into the emerging and final themes within that concept (see Multimedia Appendix 2).

Analysis will focus on detecting the key concepts among studies. The concepts will then be synthesized and refined to determine core themes; directed content analysis and thematic analysis will be undertaken to classify the data [29-31]. It is also anticipated that this multi-layer synthesis will identify novel concepts not suggested by the individual studies. Using this approach, we will identify research available in this area, the gaps in literature, and whether there is a need for a systematic review of the literature or other future reviews. Need for a systematic review will be determined by the content and methods of the studies and whether they are conducive to performing a systematic review, as the topic is very broad.

The results will then be reported in descriptive tables, frequency tables, and diagrams. The characteristics of the studies, including participants, study setting, study design, and study outcomes will be described. A summary table will also provide the identified themes.

### Knowledge-User Consultation

To ensure applicability, usability, and a clear purpose, the review is being conducted using an integrated knowledge translation approach and knowledge-users will be involved throughout the review’s duration. This approach will involve a series of consultations with research experts and the community advisory committee in our team to engage them in the development of the study outcome, action plan and research agenda, and to provide opportunities for knowledge exchange. The team includes researchers, educators, PLHIV, policymakers, clinicians, and trainees.

Preliminary findings of the review will be shared with our team, to validate our findings and guide the review’s completion, on a regular basis [20]. We will present the preliminary results and list of findings in the annual meetings of the LHIV Innovation Team. All comments and feedback will be recorded and will be integrated into the study. We will also ask the LHIV Innovation Team whether they can suggest any additional issues related to PLHIV’s access to health care from the patients’ perspective, which has not yet been identified in our review. Using the final results, a summary of possible implications to practice, including the areas that may require action in the medium and longer term, will be developed. The summary will be presented in the annual meeting of the LHIV Innovation Team for brainstorming, developing the research questions, identifying appropriate strategies, and the production of an action plan and research agenda for the LHIV Innovation Team.

## Results

### Stage 2: Identifying Relevant Studies

The search terms that were selected for the literature search can be found in Multimedia Appendix 3. The literature search strategy for PubMed is outlined in Table 1 (see Multimedia Appendix 4 for search strategies for all other databases). After an extensive literature search, 20,687 articles were found using the appropriate search terms. 7829 of these articles were duplicates which left 12,858 references to be screened.

**Table 1:** The literature search strategy for PubMed and number of identified articles.

#	Searches	
1	"HIV"[Mesh] OR "HIV Infections"[Mesh] OR HIV[tiab] OR AIDS[tiab] OR "Acquired Immunodeficiency Syndrome"[tiab] OR "Human Immunodeficiency Virus"[tiab] OR "Human Immunodeficiency Viruses"[tiab] OR "Acquired Immune Deficiency Syndrome"[tiab]	
2	Satisfaction[tiab] OR satisfy[tiab] OR perspective[tiab] OR perspectives[tiab] OR attitude[tiab] OR attitudes[tiab] OR opinion[tiab] OR opinions[tiab] OR view[tiab] OR views[tiab] OR preference[tiab] OR preferences[tiab] OR experience[tiab] OR experiences[tiab] OR "Attitude to Health"[Mesh:NoExp] OR "Patient Satisfaction"[Mesh]	
3	(access[tiab] OR accessibility[tiab] OR accessible[tiab] OR barrier[tiab] OR barriers[tiab] OR facilitator[tiab] OR facilitators[tiab] OR utilize[tiab] OR utilize[tiab] OR utilization[tiab] OR use[tiab] OR utilization[tiab] OR provision[tiab] OR provide[tiab]) AND ("health services"[tiab] OR "health service" [tiab] OR "health care"[tiab] OR healthcare[tiab] OR care[tiab] OR treatment[tiab] OR therapy[tiab] OR therapies[tiab] OR service*[tiab] OR clinic*[tiab] OR "medical care"[tiab] OR "medical services"[tiab] OR program*[tiab])	
4	"Health Services Accessibility"[Mesh] OR "Health Services/utilization"[Mesh]	
5	#3 OR #4	
6	#1 AND #2 AND #5	N=5858

### Stage 3: Study Selection

**Figure 1:**
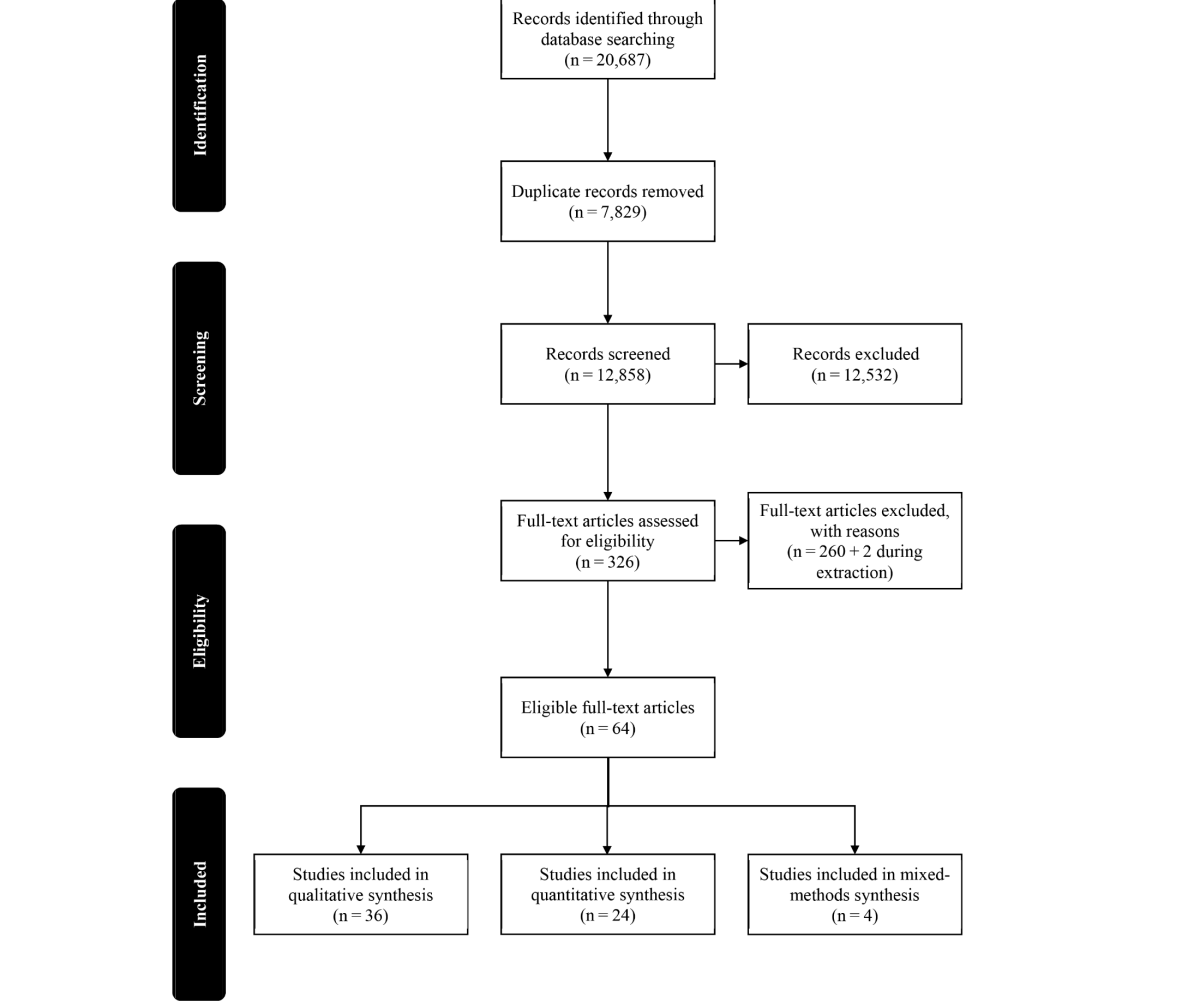
Stepwise exclusion of articles.

After assessing eligibility based on title and abstract, a total of 317 articles were deemed to be relevant (Figure 1). Of the 12,532 articles which were not relevant based on title and abstract, 700 (5%) were randomly selected to verify the results of the exclusion/inclusion process. These articles were re-assessed based on title and abstract. Of the 700, 9 articles were found to be relevant and therefore added to the 317 to be reviewed by full text. This was less than 5% of the sample and therefore verifies the process (Figure 1).

A total of 326 articles were reviewed in full text. Based on the inclusion/exclusion criteria, 260 of these articles were not about patients' perspectives on their access to care, which left 66 articles that met eligibility. A full description of the exclusion process can be found in Figure 1.

### Stage 4: Data Extraction

The final version of the data extraction tool can be viewed in Multimedia Appendix 5. During extraction, two articles were found to not be relevant to this scoping review and therefore were not included in any analysis, which left a total of 64 articles. Of these 64, 36 (56%) were qualitative, 24 (38%) were quantitative and 4 (6%) were mixed methods by design. Throughout the extraction process, a comparison of quality assessment and extraction were done by two independent reviewers. Quality assessment scores were deemed comparable if they were within 1 point of each other. If differences in extraction results were found, a discussion between the two reviewers was initiated and either a common conclusion was made, or a third party was consulted. Overall, the agreement between the two reviewers was good (ĸ=.75). The majority of assessments and extractions were similar between the reviewers, with all differences being discussed and decided upon together.

### Stage 5: Collating, Summarizing and Reporting the Results

**Figure 2:**
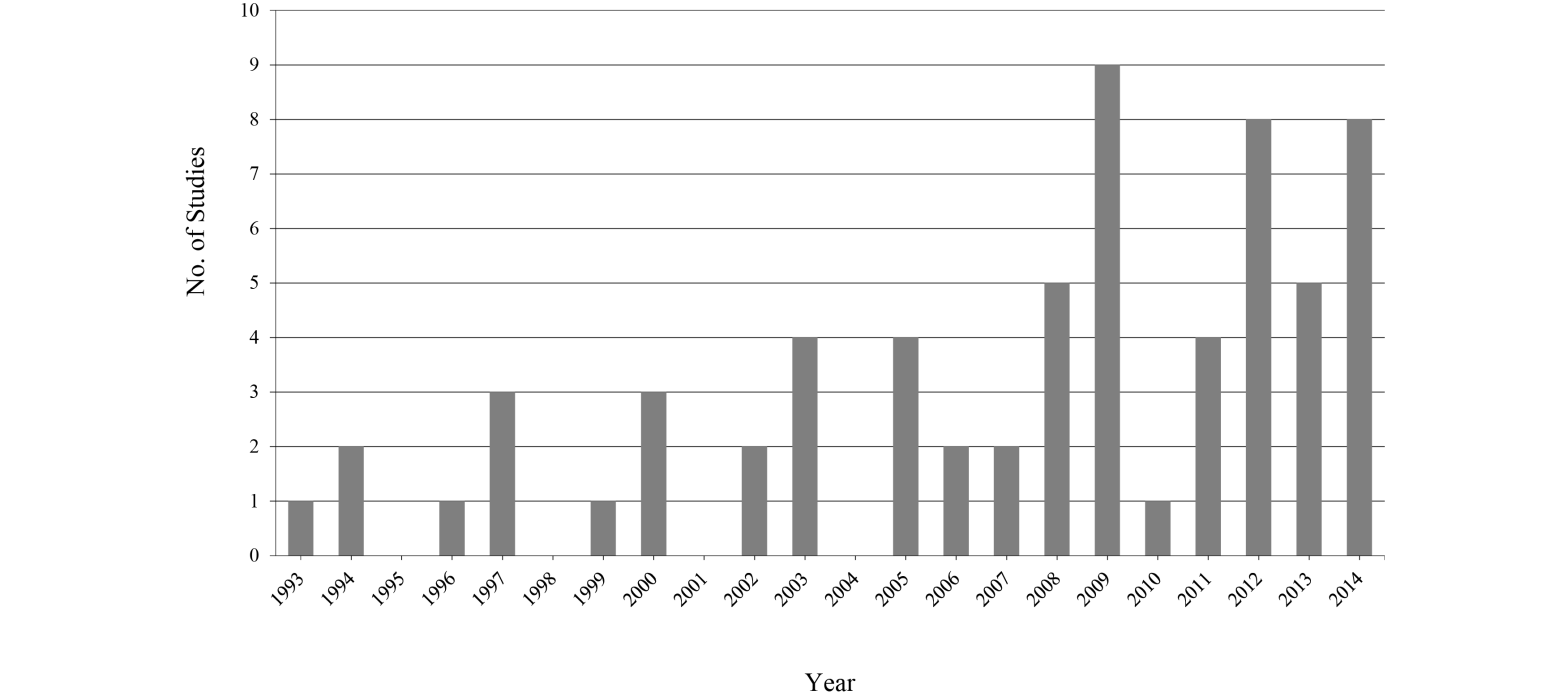
Year of publication of research.

**Figure 3:**
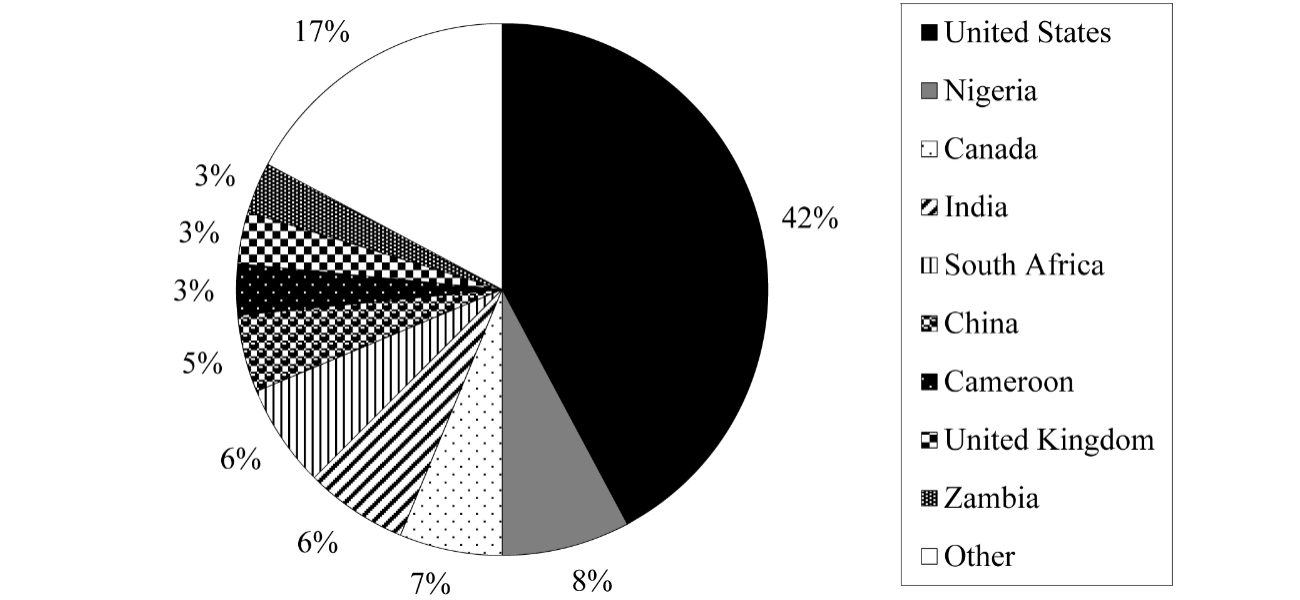
Country of study.

Looking at the articles more closely, there were some general initial quantitative results available. The majority of articles were published within the past 10 years, with the most being published in 2009 (n=9); a breakdown of all publication years can be found in Figure 2. Of the 20 different countries in which research was performed, a substantial number of the studies were conducted in the United States (n=27), with other countries such as Nigeria, Canada, India, and South Africa adding studies to the body of literature. In Figure 3, it is possible to see a visual representation of the proportion of research there has been in each country. The *other* category includes the 11 countries that are only represented in one article of this review. Data synthesis is in progress and full results are expected in June, 2016.

## Discussion

### Anticipated Challenges

A large number of search results are anticipated, so proactive steps are taken during the review, including (1) working closely with a librarian at Memorial University of Newfoundland to ensure that the review will be manageable, and (2) categorizing the studies in terms of their quality and the proper knowledge synthesis methods. We have a strong team with expertise in research methods, review methods, and content. Our team conducts regular meetings and communications with stakeholders to obtain their comments and iteratively modify the study methodology as required.

Throughout the process there have been some challenges. As there are a large number of articles identified for review, not all are initially accessible electronically through databases, and many have to be found individually. This issue adds to the workload of the research librarian, who has to manually obtain these records. Due to the wide selection of possible outcomes and the adding of new possibilities (due to an iterative process to include all aspects of patients’ perspectives of care), there may be slight variation in how results are classified between graduate students. However, the core results sections (eg, *5 A's*) remain the same, so all important results will be accounted for.

### Next Steps

Data extraction has been completed and compared by both graduate student reviewers. The next step in the process is to collate and summarize that data found, in order to answer the objectives within this study.

### Conclusion

A scoping review will record and characterize the extensive body of literature on perspectives of PLHIV regarding access to health care. Without a systematic and well-documented protocol, the scoping reviews are subject to biases. A repeatable and evidence-based protocol is required to broadly and systematically identify, classify, and synthesize literature. A valid protocol will help to identify the issues, resolve some problems, and reduce the risk of bias.

## Appendix

Multimedia Appendix 1 Additional sources of information pertaining to PLHIV.

Multimedia Appendix 2 Data summation charts.

Multimedia Appendix 3 Search terms.

Multimedia Appendix 4 Search strategies.

Multimedia Appendix 5 Data extraction tool.

## Abbreviations

AIDS: acquired immune deficiency syndrome
HIV: human immunodeficiency virus
LHIV: living with human immunodeficiency virus
MeSH: Medical Subject Heading
PLHIV: people living with human immunodeficiency virus
Tiab: title and abstract

## References

[1] Wensing M, Elwyn G (2003). Methods for incorporating patients' views in health care. BMJ.

[2] McLaughlin CG, Wyszewianski L (2002). Access to care: remembering old lessons. Health Serv Res.

[3] Siegfried N, Uthman OA, Rutherford GW (2010). Optimal time for initiation of antiretroviral therapy in asymptomatic, HIV-infected, treatment-naive adults. Cochrane Database Syst Rev.

[4] Uphold CR, Mkanta WN (2005). Review: use of health care services among persons living with HIV infection: state of the science and future directions. AIDS Patient Care STDS.

[5] The Ontario HIV Treatment Network (2014). Rapid Response Service.

[6] Carman M, Grierson J, Hurley M, Pitts M, Power J (2009). Australian Research Centre in Sex, Health & Society.

[7] Cunningham WE, Sohler NL, Tobias C, Drainoni M, Bradford J, Davis C, Cabral HJ, Cunningham CO, Eldred L, Wong MD (2006). Health services utilization for people with HIV infection: comparison of a population targeted for outreach with the U.S. population in care. Med Care.

[8] Cunningham CO, Sohler NL, McCoy K, Heller D, Selwyn PA (2005). Health care access and utilization patterns in unstably housed HIV-infected individuals in New York City. AIDS Patient Care STDS.

[9] Helleberg M, Engsig FN, Kronborg G, Larsen CS, Pedersen G, Pedersen C, Gerstoft J, Obel N (2012). Retention in a public healthcare system with free access to treatment: a Danish nationwide HIV cohort study. AIDS.

[10] Van Berckelaer A, DiRocco D, Ferguson M, Gray P, Marcus N, Day S (2012). Building a patient-centered medical home: obtaining the patient's voice. J Am Board Fam Med.

[11] Cleary PD, Edgman-Levitan S (1997). Health care quality: incorporating consumer perspectives. JAMA.

[12] Kairy D, Tousignant M, Leclerc N, Côté A, Levasseur M, Researchers TT (2013). The patient's perspective of in-home telerehabilitation physiotherapy services following total knee arthroplasty. Int J Environ Res Public Health.

[13] Van Berckelaer A, DiRocco D, Ferguson M, Gray P, Marcus N, Day S (2012). Building a patient-centered medical home: obtaining the patient's voice. J Am Board Fam Med.

[14] Gagnon M, Lepage-Savary D, Gagnon J, St-Pierre M, Simard C, Rhainds M, Lemieux R, Gauvin F, Desmartis M, Légaré F (2009). Introducing patient perspective in health technology assessment at the local level. BMC Health Serv Res.

[15] Gauvin FP, Abelson J, Giacomini M, Eyles J, Lavis JN (2010). It all depends: conceptualizing public involvement in the context of health technology assessment agencies. Soc Sci Med.

[16] Cayton H (2004). Patient and public involvement. J Health Serv Res Policy.

[17] Gulliford M, Figueroa-Munoz J, Morgan M, Hughes D, Gibson B, Beech R, Hudson M (2002). What does 'access to health care' mean?. J Health Serv Res Policy.

[18] Bryan C, Boren SA (2008). The use and effectiveness of electronic clinical decision support tools in the ambulatory/primary care setting: a systematic review of the literature. Inform Prim Care.

[19] Arksey H, O'Malley L (2005). Scoping studies: towards a methodological framework. Int J Soc Res Methodol.

[20] Kastner M, Tricco AC, Soobiah C, Lillie E, Perrier L, Horsley T, Welch V, Cogo E, Antony J, Straus SE (2012). What is the most appropriate knowledge synthesis method to conduct a review? Protocol for a scoping review. BMC Med Res Methodol.

[21] Levac D, Colquhoun H, O'Brien KK (2010). Scoping studies: advancing the methodology. Implement Sci.

[22] Moher D, Liberati A, Tetzlaff J, Altman DG (2009). Preferred reporting items for systematic reviews and meta-analyses: the PRISMA statement. BMJ.

[23] Pluye P, Gagnon MP, Griffiths F, Johnson-Lafleur J (2009). A scoring system for appraising mixed methods research, and concomitantly appraising qualitative, quantitative and mixed methods primary studies in Mixed Studies Reviews. Int J Nurs Stud.

[24] Haggerty J, Burge F, Lévesque JF, Gass D, Pineault R, Beaulieu MD, Santor D (2007). Operational definitions of attributes of primary health care: consensus among Canadian experts. Ann Fam Med.

[25] Haggerty JL, Reid RJ, Freeman GK, Starfield BH, Adair CE, McKendry R (2003). Continuity of care: a multidisciplinary review. BMJ.

[26] ProQuest (2014). Refworks.

[27] (2014). Roy Rosenzweig Center for History and New Media.

[28] Mendeley Ltd (2014). Mendeley.

[29] Walsh D, Downe S (2005). Meta-synthesis method for qualitative research: a literature review. J Adv Nurs.

[30] Jones ML (2004). Application of systematic review methods to qualitative research: practical issues. J Adv Nurs.

[31] Evans D, Pearson A (2001). Systematic reviews of qualitative research. Clin Eff Nurs.

