# Supplementary material for: Perspectives of People Living with HIV on Access to Health Care: Protocol for a Scoping Review
Source: JMIR Res Protoc. 2016 May 18;5(2):e71. doi: 10.2196/resprot.5263 (PMC4889870; doi:10.2196/resprot.5263)
Supplement: Multimedia Appendix 1 [file resprot_v5i2e71_app1.pdf]

Additional sources of information pertaining to PLHIV.

Annual Advanced Management Issues in HIV Medicine	*Organizations Relevant to HIV*	*Grey Literature on HIV*
Conference on Peer Education, Sexuality, HIV & AIDS	World Health Organization	Clinical Trials.gov
International AIDS Conference (AIDS 2014)	UNAIDS	U.K. Department of Health
Southern African HIV Clinicians Society Conference 2014	CDC	Thomson Center watch
International Conference on HIV/AIDS, STDs & STIs - 2014	AIDS.gov	TRIP (published literature would be retrieved as well)
Annual National Conference on Social Work and HIV/AIDS	AIDS Committee of Newfoundland and Labrador	
U.S. Conference on AIDS	AIDS.org	
Association of Nurses in AIDS Care 2014	AIDS Vancouver	
IAS Conference on HIV Pathogenesis, Treatment and Prevention	World AIDS Day	
Annual Canadian Conference on HIV/AIDS Research	Ontario HIV Treatment Network	
American Conference for the Treatment of HIV	HIV Edmonton	
Conference on Retroviruses and Opportunistic Infections	Regional HIV/AIDS Connection	
Infectious Diseases Society of America (IDSA) Conference	Stop HIV/AIDS	
National HIV Prevention Conference		
International Workshop on HIV Treatment, Pathogenesis and Prevention Research in Resource-poor Settings		
International Workshop on HIV & Hepatitis Co-infection		
International workshop on HV Pediatrics		

International Workshop on HIV & Aging

International Workshop on HIV Transmission – Principles of Intervention
