# Supplementary material for: Perspectives of People Living with HIV on Access to Health Care: Protocol for a Scoping Review
Source: JMIR Res Protoc. 2016 May 18;5(2):e71. doi: 10.2196/resprot.5263 (PMC4889870; doi:10.2196/resprot.5263)
Supplement: Multimedia Appendix 2 [file resprot_v5i2e71_app2.pdf]

Overview of included studies (All papers summarized)

Study author	Year	Place	Publication type	Language	Methods	Research focus	*Study goal*	*QA[1]-R1*	*QA[1]-R2*

[1]Quality Assessment, R= Reviewer

Summary of initial concepts, emerging themes and final themes (Qualitative papers summarized)

Initial concept	Relevant Papers	Emerging Themes	Final Themes

Summary of findings (Quantitative papers summarized)

**Study Author**	**Study Design**	**Country of study**	**Study Setting**	**Type of Service**	**Service Provider**	**Study Population**	**Sample Size**	**Data Collection**	**Acceptability**	**Accessibility**	**Accommodation**	**Affordability**	**Availability**	**Barriers**	**Communication**	**Preferences**	**Satisfaction**	**Equity in Access**
