# Supplementary material for: Perspectives of People Living with HIV on Access to Health Care: Protocol for a Scoping Review
Source: JMIR Res Protoc. 2016 May 18;5(2):e71. doi: 10.2196/resprot.5263 (PMC4889870; doi:10.2196/resprot.5263)
Supplement: Multimedia Appendix 3 [file resprot_v5i2e71_app3.pdf]

Search Terms for HIV:

- "HIV"[Mesh]
- "HIV Infections"[Mesh]
- HIV
- AIDS
- "Acquired Immunodeficiency Syndrome”
- "Human Immunodeficiency Virus”
- "Human Immunodeficiency Viruses"
- "Acquired Immune Deficiency Syndrome"

Search Terms for Perspective (variations of terms were also searched):

- "Attitude to Health"[Mesh:NoExp]
- "Patient Satisfaction"[Mesh]
- Satisfaction
- Perspective
- Attitude
- Opinion
- View
- Preference
- Experience

Search Terms for Access to Healthcare (variations of terms were also searched):

- "Health Services Accessibility"[Mesh]
- "Health Services/utilization"[Mesh]
- Access
- Accessibility
- Barrier
- Facilitator
- Utilize
- Use
- Provision
- "Health Services”
- "Health Care”
- Care
- Treatment
- Therapy
- Service
- Clinic
- "Medical Care”
- "Medical Services”
- Program
