# Supplementary material for: Perspectives of People Living with HIV on Access to Health Care: Protocol for a Scoping Review
Source: JMIR Res Protoc. 2016 May 18;5(2):e71. doi: 10.2196/resprot.5263 (PMC4889870; doi:10.2196/resprot.5263)
Supplement: Multimedia Appendix 4 [file resprot_v5i2e71_app4.pdf]

EMBASE Search Strategy

#	Searches	Results
1	'human immunodeficiency virus'/exp OR 'human immunodeficiency virus infection'/exp	
2	hiv:ti OR hiv:ab OR aids:ti OR aids:ab OR 'acquired immunodeficiency syndrome':ti OR 'acquired immunodeficiency syndrome':ab OR 'human immunodeficiency virus':ti OR'human immunodeficiency virus':ab OR 'human immunodeficiency viruses':ti OR 'human immunodeficiency viruses':ab OR 'acquired immune deficiency syndrome':ti OR 'acquired immune deficiency syndrome':ab	
3	#1 OR #2	
4	'attitude to health'/exp OR 'patient attitude'/de OR 'patient preference'/exp OR 'patient satisfaction'/exp	
5	satisfaction:ti OR satisfy:ti OR perspective*:ti OR attitude*:ti OR opinion*:ti OR view*:ti OR preference*:ti OR experience*:ti OR satisfaction:ab OR satisfy:ab OR perspective*:ab ORattitude*:ab OR opinion*:ab OR view*:ab OR preference*:ab OR experience*:ab	
6	#4 OR #5	
7	(access*:ti OR barrier*:ti OR facilitator*:ti OR utiliz*:ti OR utilis*:ti OR provision:ti OR provide:ti OR access*:ab OR barrier*:ab OR facilitator*:ab OR utiliz*:ab OR utilis*:ab OR provision:ab OR provide:ab) AND ('health service':ti OR 'health services':ti OR 'health care':ti OR healthcare:ti OR care:ti OR treatment:ti OR therapy:ti OR therapies:ti OR service*:ti OR clinic*:ti OR 'medical care':ti OR 'medical services':ti OR program*:ti OR 'health service':ab OR 'health services':ab OR 'health care':ab OR healthcare:ab OR care:ab OR treatment:ab OR therapy:ab OR therapies:ab OR service*:ab OR clinic*:ab OR 'medical care':ab OR 'medical services':ab OR program*:ab)	

8	'health care utilization'/exp	
9	#7 OR #8	
10	#3 AND #6 AND #9	8,426

CINAHL Search Strategy

#	Searches	Results
1	(MH "HIV Infections+") OR (MH "Human Immunodeficiency Virus+") OR (MH "HIV- Infected Patients+")	
2	TI (HIV OR AIDS OR "acquired immunodeficiency syndrome" OR "human immunodeficiency virus" OR "human immunodeficiency viruses" OR "acquired immune deficiency syndrome") OR AB (HIV OR AIDS OR "acquired immunodeficiency syndrome" OR "human immunodeficiency virus" OR "human immunodeficiency viruses" OR "acquired immune deficiency syndrome")	
3	#1 OR #2	
4	(MH "Attitude to Health") OR (MH "Health Beliefs") OR (MH "Patient Satisfaction")	
5	TI (satisfaction OR satisfy OR perspective* OR attitude* OR opinion* OR view* OR preference* OR experience*) OR AB (satisfaction OR satisfy OR perspective* OR attitude* OR opinion* OR view* OR preference* OR experience*)	
6	#4 OR #5	
7	(MH "Health Services Accessibility+") OR (MH "Health Resource Utilization") OR (MH "Health Services+/UT")	
8	TI (access* OR barrier* OR facilitator* OR utiliz* OR utilis* OR provision OR provide) OR AB (access* OR barrier* OR	

	facilitator* OR utiliz* OR utilis* OR provision OR provide)	
9	TI ("health services" OR "health service" OR "health care" OR healthcare OR care OR treatment OR therapy OR therapies OR service* OR clinic* OR "medical care" OR "medical services" OR program*) OR AB ("health services" OR "health service" OR "health care" OR healthcare OR care OR treatment OR therapy OR therapies OR service* OR clinic* OR "medical care" OR "medical services" OR program*)	
10	#8 AND #9	
11	#7 OR #10	
12	#3 AND #6 AND #11	2,792

PsycINFO Search Strategy

#	Searches	Results
1	DE "HIV" OR DE "AIDS"	
2	TI (HIV OR AIDS OR "acquired immunodeficiency syndrome" OR "human immunodeficiency virus" OR "human immunodeficiency viruses" OR "acquired immune deficiency syndrome") OR AB (HIV OR AIDS OR "acquired immunodeficiency syndrome" OR "human immunodeficiency virus" OR "human immunodeficiency viruses" OR "acquired immune deficiency syndrome")	
3	#1 OR #2	
4	DE "Client Satisfaction"	
5	TI (satisfaction OR satisfy OR perspective* OR attitude* OR opinion* OR view* OR preference* OR experience*) OR AB (satisfaction OR satisfy OR perspective* OR attitude* OR opinion* OR view* OR preference* OR experience*)	

6	#4 OR #5	
7	DE "Health Care Utilization"	
8	DE "Health Care Services" OR DE "Continuum of Care" OR DE "Long Term Care" OR DE "Mental Health Services" OR DE "Palliative Care" OR DE "Primary Health Care"	
9	TI (access* OR barrier* OR facilitator* OR utiliz* OR utilis* OR provision OR provide) OR AB (access* OR barrier* OR facilitator* OR utiliz* OR utilis* OR provision OR provide)	
10	TI ("health services" OR "health service" OR "health care" OR healthcare OR care OR treatment OR therapy OR therapies OR service* OR clinic* OR "medical care" OR "medical services" OR program*) OR AB ("health services" OR "health service" OR "health care" OR healthcare OR care OR treatment OR therapy OR therapies OR service* OR clinic* OR "medical care" OR "medical services" OR program*)	
11	#8 OR #10	
12	#9 AND #11	
13	#7 OR #12	
14	#3 AND #6 AND #13	2781

Cochrane Search Strategy

#	Searches	Results
1	MeSH descriptor: [Health Services Accessibility] explode all trees	
2	MeSH descriptor: [Health Services] explode all trees and with qualifier(s): [Utilization - UT]	
3	(access* or barrier* or facilitator* or utiliz* or utilis* or provision or provide) and ("health services" or "health	

	service" or "health care" or healthcare or care or treatment or therapy or therapies or service* or clinic* or "medical care" or "medical services" or program*):ti,ab,kw (Word variations have been searched)	
4	MeSH descriptor: [Attitude to Health] this term only	
5	MeSH descriptor: [Patient Satisfaction] explode all trees	
6	satisfaction or satisfy or perspective* or attitude* or opinion* or view or views or preference* or experience*:ti,ab,kw (Word variations have been searched)	
7	MeSH descriptor: [HIV] explode all trees	
8	MeSH descriptor: [HIV Infections] explode all trees	
9	HIV or AIDS or "acquired immunodeficiency syndrome" or "human immunodeficiency virus" or "human immunodeficiency viruses" or "acquired immune deficiency syndrome":ti,ab,kw (Word variations have been searched)	
10	#1 or #2 or #3	
11	#4 or #5 or #6	
12	#7 or #8 or #9	
13	#10 AND #11 AND #12	830
