# Supplementary material for: Perspectives of People Living with HIV on Access to Health Care: Protocol for a Scoping Review
Source: JMIR Res Protoc. 2016 May 18;5(2):e71. doi: 10.2196/resprot.5263 (PMC4889870; doi:10.2196/resprot.5263)
Supplement: Multimedia Appendix 5 [file resprot_v5i2e71_app5.pdf]

A. Review Details		Notes
**Reference:**		
**Refid Number:**		
**First Author**		
**Year of Publication**		
**Place of Publication**		
**Peer or Grey**	Peer reviewed	
	Grey literature	
**Type of Publication**	Primary research	
	Review or meta-analysis	
	Commentary	
	Protocol	
	Conference proceeding	
	Working paper	
	Dissertation/thesis	
	Report (NGO)	
	Report (government)	
	Fact sheet	
	Policy/position statement	
	News article/ Op-ed	
	Other	
**B. Project Details**		
**Program/Project Name**		
**Country**		
**Location**		
**Geographical Setting**	Urban	
	Rural	
	Rural-remote	
	Not specified/Not reported	
**Site of Study**	Aboriginal health/ friendship centres	
	Community health centre	
	Dental Clinic	
	General Healthcare	
	Home Care	
	Hospital	
	Outpatient	
	Government ART Center	
	Primary care clinic/ Family health clinic	
	Prisons/Correctional Facilities	
	Sexual health/HIV Clinic	
	Other	
	Not specified/Not reported	
**Duration of study**	less than one month	
	1-3 months	
	4-6 months	
	7-11 months	
	1-2 years	
	3-5 years	
	6 years +	
	Not specified/Not reported	
**C. Sample Characterisitcs**		
**Age**		
**Gender**	Cisgender Male	
	Cisgender Female	
	Transgender male	
	Transgender female	
	Two-spirited	
	Other	
	Not specified/Not reported	
**Sub-community**	MSM	

	LGBTQ<br>PWID or people who smoke crack<br>People who smoke crack<br>Aboriginal peoples<br>Racialized groups<br>Transgender individuals<br>Commercial sex workers<br>Inmates<br>People born outside of Canada<br>Pregnant women<br>People receiving ART<br>Other<br>Not specified/Not reported
**Participant health status**	Excellent<br>Very good<br>Good<br>Fair<br>Poor<br>Other<br>Not specified/Not reported
**D. Methodology**	
**Aim of study**	Access to Care<br>Satisfaction with Care<br>Barriers to Care<br>Barriers to ART<br>Other<br>Not specified/Not reported
**Study approach**	Quantitative<br>Qualitative<br>Mixed-methods
**Study design**	RCT<br>Non-randomized control trial<br>Case report<br>Case series<br>Case-control<br>Cross-sectional<br>Cohort study<br>Correlation study<br>Ethnography<br>Grounded theory<br>Narrative research<br>Phenomenological research<br>Observational<br>Community based research design<br>Systematic reviews<br>Meta-analysis<br>Scoping reviews<br>Field trials<br>Other
**Data collection method**	Interview<br>Survey<br>Focus group<br>Observation<br>Case study<br>Document review/analysis<br>Other
**Sampling**	Non-probability<br>Probability
**Sample Size**	
**Participation Rate**	
**Tool**	
**E. Findings**	
**Type of Service**	Pharmaceutical<br>Primary care<br>Clinical/curative<br>Dental<br>Preventative<br>Outpatient

	Inpatient<br>Social<br>Promotive<br>Rehabilitative<br>General Health Care<br>Mental Health Care<br>Not reported<br>Other
**Compensation**	Yes<br>No<br>Not applicable/ not reported
**Service Provider**	Doctor<br>Nurse<br>Dentist<br>Dental Assistant<br>Pharmacist<br>Outreach Worker<br>Social Worker<br>Psychologist<br>Other<br>Not specified/Not reported
**Outcome Measures**	**Acceptability**<br>***Acceptability - Supply***<br>Complexity of billing system<br>Staff interpersonal skills<br>Technology<br>Characteristics of the health services<br>Other<br>***Acceptability -Demand***<br>Households' expectations<br>Low self-esteem and little assertiveness<br>Community and cultural preferences, attitudes and norms<br>Stigma<br>Health awareness<br>User's attitudes and expectations<br>Trust, feel welcomed<br>Feel Known<br>Other<br>**Accessibility**<br>***Perceived Access***<br>Gaining entry into the health care system.<br>Having a regular doctor<br>Continuity of care<br>services<br># of visits/# services used<br>Other<br>***Accessibility -Supply***<br>Service location<br>Other<br>***Accessibility -Demand***<br>Indirect costs to household (transport)<br>Means of transport available<br>Other<br>**Accommodation/Adequacy**<br>***Accommodation/Adequacy- Supply***<br>Opening hours<br>Other<br>***Accommodation/Adequacy -Demand***<br>Clean and well-kept facility<br>Appointment scheduling<br>Other<br>**Affordability**<br>***Affordability -Supply***<br>Costs and prices of services, including informal payments<br>Private–public dual practices<br>Other<br>***Affordability -Demand***<br>Household resources and willingness to pay<br>Opportunity costs

	Cash flow within society<br>Other<br>**Availability**<br>***Availability- Supply***<br>Unqualified health workers,<br>Staff absenteeism<br>Waiting time<br>Motivation of staff<br>Drugs and other consumable<br>Non-integration of health services<br>Lack of opportunity (exclusion from services)<br>Late or no referral<br>Other<br>***Availability -Demand***<br>Information on health care services/providers<br>Education<br>Other<br>**Barriers**<br>Physical<br>Financial<br>Sociocultural<br>Organizational<br>Personal<br>Health/Medical reasons<br>Other<br>**Communication**<br>Person-centeredness<br>Patient-provider relationship<br>Patient Empowerment<br>**Preferences**<br>**Satisfaction**<br>**Equity in Access**<br>**Patient Demographics**<br>**Other**
**Significant outcomes-quantitative**	**Acceptability**<br>***Acceptability- Supply***<br>High complexity of billing system<br>Low complexity of billing system<br>Staff interpersonal skills- High<br>Staff interpersonal skills- Low<br>Has the appropriate technology<br>Does not have the appropriate technology<br>Data correlates- Characteristics of the health services<br>Other<br>***Acceptability -Demand***<br>Met Households' expectations<br>Did not meet households' expectations<br>Low self-esteem and little assertiveness<br>Community and cultural preferences, attitudes and norms<br>Stigma- High<br>Stigma- Low<br>High health awareness<br>Lack of health awareness<br>User's attitudes and expectations<br>Trust, feel welcomed- High<br>Trust, feel welcomed- Low<br>Feel known- High<br>Feel known- Low<br>Other<br>**Accessibility**<br>***Perceived Access***<br>Gaining entry into the health care system- Easy<br>Gaining entry into the health care system- Hard<br>Having a regular doctor<br>High continuity of care<br>low continuity of care<br>access to sites of care for needed services- Easy<br>access to sites of care for needed services- Hard<br># of visits/# services used

Other
Accessibility -Supply
Service location- Good
Service location- Poor
Other
Accessibility -Demand
Indirect costs to household (transport)- High
Indirect costs to household (transport)- Low
Means of transport available
Other
Accommodation/Adequacy
Accommodation/Adequacy- Supply
Opening hours- Good
Opening hours- Poor
Other
Accommodation/Adequacy -Demand
Clean and well-kept facility
Appointment scheduling- Good
Appointment scheduling- Poor
Other
Affordability
Affordability -Supply
Costs and prices of services, including informal payments- High
Costs and prices of services, including informal payments- Low
Private–public dual practices
Other
Affordability -Demand
Household resources and willingness to pay- High
Household resources and willingness to pay- Low
Opportunity costs- High
Opportunity costs- Low
Cash flow within society- High
Cash flow within society- Low
Other
Availability
Availability- Supply
Unqualified health workers
Staff absenteeism
Waiting time- High
Waiting time- Low
Motivation of staff- High
Motivation of staff- Low
Drugs and other consumable- often available
Drugs and other consumable- hardly ever available
Non-integration of health services
Lack of opportunity (exclusion from services)
Late or no referral
Other
Availability -Demand
Information on health care services/providers- High
Information on health care services/providers- Low
Education-High
Education-Low
Other
Barriers
Physical (eg. Geographic, transportation ect.)
support ect.)
of support, stigma ect.)
Organizational (policies, procedures ect.)
Personal (active drug use, low motivation, other conditions)
Health/Medical reasons
Other
Communication (general)- High
Communication(general)- Low
Person-centeredness- High
Person-centeredness- Low
Patient-provider relationship- Good
Patient-provider relationship- Bad
Patient Empowerment- High

	Patient Empowerment- Low
	**Preferences of care**
	**Satisfaction with Services/Care- High**
	**Satisfaction with Services/Care- Low**
	**Equity in Access- Good**
	**Equity in Access- Poor**
	**Patient Demographics**
	**Other**
	Not reported
**Qualitative and non-significant outcomes**	**Acceptability**
	Acceptability- Supply
	High complexity of billing system
	Low complexity of billing system
	Staff interpersonal skills- High
	Staff interpersonal skills- Low
	Has the appropriate technology
	Does not have the appropriate technology
	Data correlates- Characteristics of the health services
	Other
	Acceptability -Demand
	Met Households' expectations
	Did not meet households' expectations
	Low self-esteem and little assertiveness
	Community and cultural preferences, attitudes and norms
	Stigma- High
	Stigma- Low
	High health awareness
	Lack of health awareness
	User's attitudes and expectations
	Trust, feel welcomed- High
	Trust, feel welcomed- Low
	Feel known- High
	Feel known- Low
	Other
	**Accessibility**
	Perceived Access
	Gaining entry into the health care system- Easy
	Gaining entry into the health care system- Hard
	Having a regular doctor
	High continuity of care
	low continuity of care
	access to sites of care for needed services- Easy
	access to sites of care for needed services- Hard
	# of visits/# services used
	Other
	Accessibility -Supply
	Service location- Good
	Service location- Poor
	Other
	Accessibility -Demand
	Indirect costs to household (transport)- High
	Indirect costs to household (transport)- Low
	Means of transport available
	Other
	**Accommodation/Adequacy**
	Accommodation/Adequacy- Supply
	Opening hours- Good
	Opening hours- Poor
	Other
	Accommodation/Adequacy -Demand
	Clean and well-kept facility
	Appointment scheduling- Good
	Appointment scheduling- Poor
	Other
	**Affordability**
	Affordability -Supply
	Costs and prices of services, including informal payments- High
	Costs and prices of services, including informal payments- Low
	Private–public dual practices

	Other<br>***Affordability -Demand***<br>Household resources and willingness to pay- High<br>Household resources and willingness to pay- Low<br>Opportunity costs- High<br>Opportunity costs- Low<br>Cash flow within society- High<br>Cash flow within society- Low<br>Other<br>**Availability**<br>***Availability- Supply***<br>Unqualified health workers<br>Staff absenteeism<br>Waiting time- High<br>Waiting time- Low<br>Motivation of staff- High<br>Motivation of staff- Low<br>Drugs and other consumable- often available<br>Drugs and other consumable- hardly ever available<br>Non-integration of health services<br>Lack of opportunity (exclusion from services)<br>Late or no referral<br>Other<br>***Availability -Demand***<br>Information on health care services/providers- High<br>Information on health care services/providers- Low<br>Education-High<br>Education-Low<br>Other<br>**Barriers**<br>Physical (eg. Geographic, transportation ect.)<br>support ect.)<br>of support, stigma ect.)<br>Organizational (policies, procedures ect.)<br>Personal (active drug use, low motivation, other conditions)<br>Health/Medical reasons<br>Other<br>**Communication (general)- High**<br>**Communication(general)- Low**<br>Person-centeredness- High<br>Person-centeredness- Low<br>Patient-provider relationship- Good<br>Patient-provider relationship- Bad<br>Patient Empowerment- High<br>Patient Empowerment- Low<br>**Preferences of care**<br>**Satisfaction with Services/Care- High**<br>**Satisfaction with Services/Care- Low**<br>**Equity in Access- Good**<br>**Equity in Access- Poor**<br>**Patient Demographics**<br>**Other**<br>Not reported
**Conclusions**	
**Limitations**	
